# Effects of a new thyrotropic drug isolated from *Potentilla alba* on the male reproductive system of rats and offspring development

**DOI:** 10.1186/s12906-020-03184-z

**Published:** 2021-01-13

**Authors:** Lubov V. Krepkova, Valentina V. Bortnikova, Aleksandra N. Babenko, Praskovya G. Mizina, Vladimir A. Mkhitarov, Kathleen M. Job, Catherine M. Sherwin, Elena Y. Enioutina

**Affiliations:** 1 Center of Medicine, All-Russian Research Institute of Medicinal and Aromatic Plants (VILAR), Grina Street 7, Moscow, 117216 Russia; 2 FSBI “Research Institute of Human Morphology”, 3 Tsyurupy St., Moscow, 117418 Russia; 3 Division of Clinical Pharmacology, Department of Pediatrics, University of Utah School of Medicine, 295 Chipeta Way, Salt Lake City, UT 84108 USA; 4 Department of Pediatrics, Wright State University, Boonshoft School of Medicine, Dayton Children’s Hospital, Children’s Plaza, Dayton, OH 45404 USA; 5 Department of Pharmaceutics & Pharmaceutical Chemistry, College of Pharmacy, University of Utah, 30 South 2000 East, Salt Lake City, UT 84112 USA

**Keywords:** Herbal drug toxicity, Male reproductive health, Preclinical drug evaluation, *Potentilla alba*

## Abstract

**Background:**

The dysfunction of the thyroid gland is a common medical condition. Nowadays, patients frequently use medicinal herbs as complementary or alternative options to conventional drug treatments. These patients may benefit from treatment of thyroid dysfunctions with *Potentilla alba* L. preparations. While it has been reported that *Potentilla alba* preparations have low toxicity, nothing is known about their ability to affect reproductive functions in patients of childbearing age.

**Methods:**

Male Wistar rats were orally treated with a thyrotrophic botanical drug, standardized *Potentilla alba* Dry Extract (PADE), at doses 8 and 40 times higher than the median therapeutic dose recommended for the clinical trials, for 60 consecutive days. Male Wistar rats receiving water (H_2_O) were used as controls. After completing treatment, half of the PADE-treated and control males were used to determine PADE gonadotoxicity, and the remaining half of PADE-treated and control males were mated with intact females. Two female rats were housed with one male for two estrus cycles. PADE effects on fertility and fetal/offspring development were evaluated.

**Results:**

Herein, we report that oral treatment of male Wistar rats with PADE before mating with intact females instigated marked effects on male reproductive organs. Treatment significantly decreased the motility of the sperm and increased the number of pathological forms of spermatozoa. Additionally, a dose-dependent effect on Leydig cells was observed. However, these PADE effects did not significantly affect male fertility nor fetal and offspring development when PADE-treated males were mated with intact females.

**Conclusions:**

PADE treatment of male rates negatively affected sperm and testicular Leydig cell morphology. However, these changes did not affect male fertility and offspring development. It is currently not known whether PADE treatment may affect human male fertility and offspring development. Therefore, these results from an animal study need to be confirmed in humans. Results from this animal study can be used to model the exposure-response relationship and adverse outcomes in humans.

## Background

Thyroid gland dysfunction is a common medical condition [1–3]. Nowadays, many patients choose to use medicinal herbs and herb-based medications as an alternative or complementary treatment for various diseases and conditions including thyroid dysfunctions. Various medicinal herbs affect thyroid gland function. Gotu Kola (*Centella asiatica*, L.), Ashwagandha (*Withania somnifera* (L.) Dunal), and Guggul (*Commiphora mukul* (Arn.) Bhandari) are effective for the treatment of hypothyroidism [4]. There are also medicinal herbs that have thyroid suppressive properties and can be recommended for the treatment of hyperthyroidism: bugleweed (*Lycopus virginicus*, L.), gypsywort (*Lycopus europaeus,* L.), Lemonbalsam (*Melissa officinalis*, L.), Rosemary (*Rosmarinus officinalis*, L.) and Sage (*Salvia officinalis*, L.), and others [4].

Patients with thyroid dysfunctions may also benefit from using preparations from *Potentilla alba* L. (White cinquefoil). It has been shown that extracts prepared from the rhizomes of *Potentilla alba* have thyrotropic properties and have been used to treat patients with hypo- and hyperthyroidism [5–7]. *Potentilla alba* extracts also have demonstrated anti-inflammatory, anti-microbial, antioxidant, and adaptogenic properties [8, 9].

*Potentilla alba* L. is a member of the Potentilla genus belonging to the family Rosaceae. This perennial herbaceous plant is native to Central, Southern, and Eastern parts of Europe and introduced to the U.S. [8, 10, 11]. Phytochemical studies determined that the *Potentilla alba* plant possesses several biologically active constituents, including flavonoids, polyphenols, phenol carboxylic acids, triterpenes, tannins, and polysaccharides [8, 12].

Both males and females of childbearing age may use this plant or botanical drugs prepared from this plant. Therefore, it is essential to investigate whether *Potentilla alba* has any adverse effects on the male or female reproductive systems. Treatment of patients of childbearing age may also negatively affect fetal and offspring developments. To our knowledge, no published studies have explored the effects of *Potentilla alba* standardized extracts on the male reproductive health.

Studies presented herein investigate the effects of standardized *Potentilla alba* Dry Extract (PADE) on the reproductive system of male Wistar rats and the development of fetuses and offspring born from males treated with PADE.

## Methods

### Botanical drug

Standardized *Potentilla alba* Dry Extract was developed by the All-Russian Institute of Medicinal and Aromatic Plants (VILAR), Moscow, Russian Federation. The dry extract was prepared from the rhizomes of cultivated white cinquefoil (*Potentilla alba* L.) plants. PADE appeared as a light brown, amorphous, hygroscopic powder. PADE was soluble in 40% ethanol and formed a turbid solution in hot water. PADE preparation consists of (+)-catechin, gallic acid, p-coumaric acid, β-sitosterol, β-sitosterol-3-O-β-D- glucopyranoside, and others [13, 14]. The dry extract was standardized by the sum of phenolic compounds (e.g., catechins, gallic, and coumaric acids). The composition of PADE used in these experiments comprised of 61.29% phenolic compounds, 25% polysaccharides, and 2% phytosterols. In addition, the PADE also contained polymeric proanthocyanidins of unknown structure.

### Animals

The study of PADE’s effects on the reproductive function of male rats was carried out in accordance with the “Guidelines for conducting preclinical studies of drugs” [15]. Three to 4-month-old female (180–200 g) and male (200–250 g) Wistar rats were obtained from the animal nursery “Andreevka,” a branch of FSBIS of the Scientific Center of Biomedical Technologies of the Medical and Biological Agency, Russia. Animals were housed in the VILAR biomedical clinic for at least 3 weeks before experiment initiation. Animals were housed five rats of the same gender in a plastic cage before mating and one male and two females during mating. Animals were kept under controlled environmental conditions of light (12-h light cycle), temperature (20–22 °C), and humidity (40–60%) with free access to standard chow and water. At the end of each experiment, adult animals and offspring were euthanized by CO_2_ inhalation. Feti were considered to be euthanized after confirmed euthanasia and death of the mother. The study protocol has been approved by the VILAR Ethical Committee on Animal Experimentation and carried out per the “European Convention for the Protection of Vertebrate Animals Used for Experimental and other Scientific Purposes (ETS 123). Strasbourg, 1986”. The study evaluating effects of PADE on male rat reproductive health were conducted under “Guidelines for conducting preclinical studies of drugs” implemented by the Ministry of Public Health of the Russian Federation [16].

### PADE treatment

Both male and female rats were randomly assigned to either control or experiment groups. Freshly prepared 2.5% aqueous PADE solution was administered every morning to male rats by gavage at doses of 25 mg/kg and 125 mg/kg for 60 consecutive days prior to mating. The low PADE dose represented a dose approximately equal to median effective dose for rats (20 mg/kg, ED_50_) and 8 times higher than median therapeutic dose recommended for clinical trials (3.1 mg/kg). High dose administered to experimental animals was 40 times higher than therapeutic recommended for clinical trials. The dose of the extract was calculated based on body weight, which was determined when the animal was last weighted. The animals were weighed once a week. Male rats receiving water (H_2_O) were used as controls. Twenty to thirty male rats were used in each group. After completing the treatment, half of PADE-treated and control males were used to determine PADE gonadotoxicity, and the remaining half of PADE-treated and control males were mated with intact females. Two female rats were housed with one male for two estrus cycles. Vaginal smears were evaluated daily for the estrus cycle and the presence of sperm. At the time of insemination, females were separated from males. All experiments described in this study were repeated twice.

### Investigation of PADE effects on male fertility

The effects of PADE treatment on male fertility were evaluated by measuring fertility and pregnancy indexes. The fertility index was calculated as following the number of pregnant rats divided by the number of fertilized rats and multiplied by 100. The pregnancy index was calculated as a ratio between the number of delivered dams and the number of pregnant dams multiplied by 100.

### Assessment of PADE effects on the male reproductive system

Evaluation of the gonadotoxic effects of PADE was conducted the day after treatment completion. The functional conditions of spermatozoa were evaluated by assessing sperm motility and the percentage of pathologic forms. Sperm was obtained immediately after euthanizing rats by vortexing a longitudinally cut-open epididymis in 10 ml of 0.9% sodium chloride (NaCl) for 2 min. The sperm suspension was placed into the hemocytometer. Total and motile spermatozoon numbers were counted. A minimum of 200 spermatozoa per animal was counted. Sperm motility was presented as a percentage of motile sperm in a sample.

Additionally, sperm motility time was determined. A drop of sperm suspension was placed onto a slide and kept in a humid chamber at ~ 37 °C. Sperm motility was monitored every 5–7 min until complete cessation of all sperm movement.

To assess the presence of pathologic forms of spermatozoa, a small amount of sperm suspension was placed onto the histological slide, ethanol fixed for 10 min, air-dried, stained with Giemsa staining, and reviewed under a microscope (magnification 200-400x). A minimum of five view-fields was counted.

To evaluate the testicular morphology, a 10 mm testicular piece was fixed in 10% neutral formalin, embedded in paraffin, and sectioned. Ten μm sections were stained with hematoxylin and eosin. The index of spermatogenesis was used to evaluate the spermatogenic competence of the testes quantitatively. The number of spermatogenic epithelium layers was counted in 100 convoluted seminiferous tubules. The index of spermatogenesis was calculated as the following: IS = (a1*n + a2*n + a3*n + a4*n)/100, where a1*n is the number of convoluted seminiferous tubules containing one layer (spermatogonia), a2*n is the number of convoluted seminiferous tubules containing two layers (spermatogonia and spermatocytes); a3*n is the number of convoluted seminiferous tubules containing three layers (spermatogonia, spermatocytes, spermatids); and 4a*n is the number of convoluted seminiferous tubules containing four layers (spermatogonia, spermatocytes, spermatids, spermatozoa).

Morphometric analysis of Leydig cells was performed using images obtained with an Axioskope 2 microscope (Carl Zeiss Microscopy, Germany) equipped with AxioCam and digital image processing software AxioVision 4.6. The number and size of the nucleus of the Leydig cells were measured [17].

### Investigation of PADE effects on fetal and offspring development

Pregnant rats were divided into two cohorts. Cohort 1 was used to study PADE’s impact on fetal growth, and cohort 2 was used to research offspring development. Pregnant rats of cohort 1 (8–11 dams in each group) were sacrificed on day 20 of pregnancy by carbon dioxide asphyxiation. The number of corpora lutea, the status of each implant site (live/dead embryo, early/late resorption), and embryo body weight were determined. Cohort 2 (8–11 dams in each group) was left to deliver their offspring naturally. Weight gain and survival of the born offspring were assessed and recorded on day 21 after birth. The development of motor-sensory reflexes of the progeny (open field test) was evaluated on day 30 after birth. The open field apparatus consisted of an unobstructed field with walls preventing animal escape. The field is marked with black and white squares. The corners of each square had round holes. Each test animal was placed into the field for 3 min. An investigator recorded the number of squares crossed by the rat, how many times animal was rearing, grooming, defecating and head dipping into the holes.

### Statistical analysis

Data obtained from every experimental or control animal were included in the analysis. Results included male fertility, gonadotoxic effects, pre- and post-implantation death, number of newborns, and offspring postnatal development. Statistical analysis of the results was conducted using Statistica software, version 10 with the “SygmaStat 3.5” package (Systat Software Inc., CA, USA). Numeric data presented in Table 2 were analyzed using a two-way analysis of variance (ANOVA). For the comparison of experimental groups, a post hoc Dunn’s multiple comparisons test was performed.

## Results

### PADE effects on male fertility

The results presented in Table 1 demonstrate that oral treatment of male rats with 25 or 125 mg/kg (8 times and 40 times ED_50_, respectively) for 60 days before mating with intact female rats resulted in an approximately 5% decrease in fertility index. These changes, however, were not statistically significant when compared to the control group receiving H_2_O by the gavage. All fertilized female rats became pregnant.

**Table 1:** PADE effect on male fertility of Wistar rats

Parameters	Study groups^**a**^		
	***Control, H***_***2***_***O***	***PADE, 25 mg/kg***	***PADE, 125 mg/kg***
No. of mating female rats	17	18	19
Fertility index (%)	100	94.4	94.7
Pregnancy index (%)	100	100	100

^a^Male rats were treated with either H_2_O (control) or PADE (25 or 125 mg/kg) for 60 days before mating

### Effects of PADE on the male reproductive system

Repeated oral administration of PADE to male rats at a 25 mg/kg dose did not significantly affect the total number of spermatozoa compared to the control group (Table 2). Significantly reduced number of motile forms was observed (*p* < 0.05) in the PADE treated group. The spermatozoon movement’s duration decreased, but these differences were not statistically significant. A higher percent of spermatozoa pathological forms were found in the testis of male rats treated with 25 mg/kg compared with the testis of the control group. These differences were statistically significant (*p* < 0.05). Also, a morphological study of the testis of rats treated with a low dose of PADE revealed a statistically significant decrease in the spermatogenesis index compared to controls (Table 2), indicating a suppression of the spermatogenesis. The testicular weight was not affected by the treatment.

**Table 2:** PADE gonadotropic effects

Parameters	Study groups^**a**^		
	***Control***	***PADE, 25 mg/kg***	***PADE, 125 mg/kg***
Total number of spermatozoa (× 10^6^)	591.1+ 37.2	599.4 + 35.5	481.2 + 54.5
Motile spermatozoa (×10^6^)	327.8 + 18.6	240.6 + 26.6**	163.3 + 29.1**
Motile spermatozoa (%)	56.1 + 2.9	46.3 + 1.7**	32.8 + 3.3**
Duration of spermatozoon movement (min.)	170.0 + 13.5	162.5 + 16.0	98.0 + 11.4**
Pathologic forms of spermatozoa (%)	1.5+ 0.3	2.7 + 0.2**	5.1 + 0.5**
Testicular weight (g)	3.2 + 0.1	3.3 + 0.1	3.4 + 0.2
Relative testicular weight (%)	0.76 + 0.04	0.82 + 0.02	0.90 + 0.04
Spermatogenesis index, Median (range)	3.36 (3.28,3.42)	3.08 (3.04,3.11) **	3.02 (3.0,3.05) **

^a^Male rats were treated with either H_2_O (control) or PADE (25 or 125 mg/kg) for 60 days before mating; **p < 0.05

**Fig. 1:**
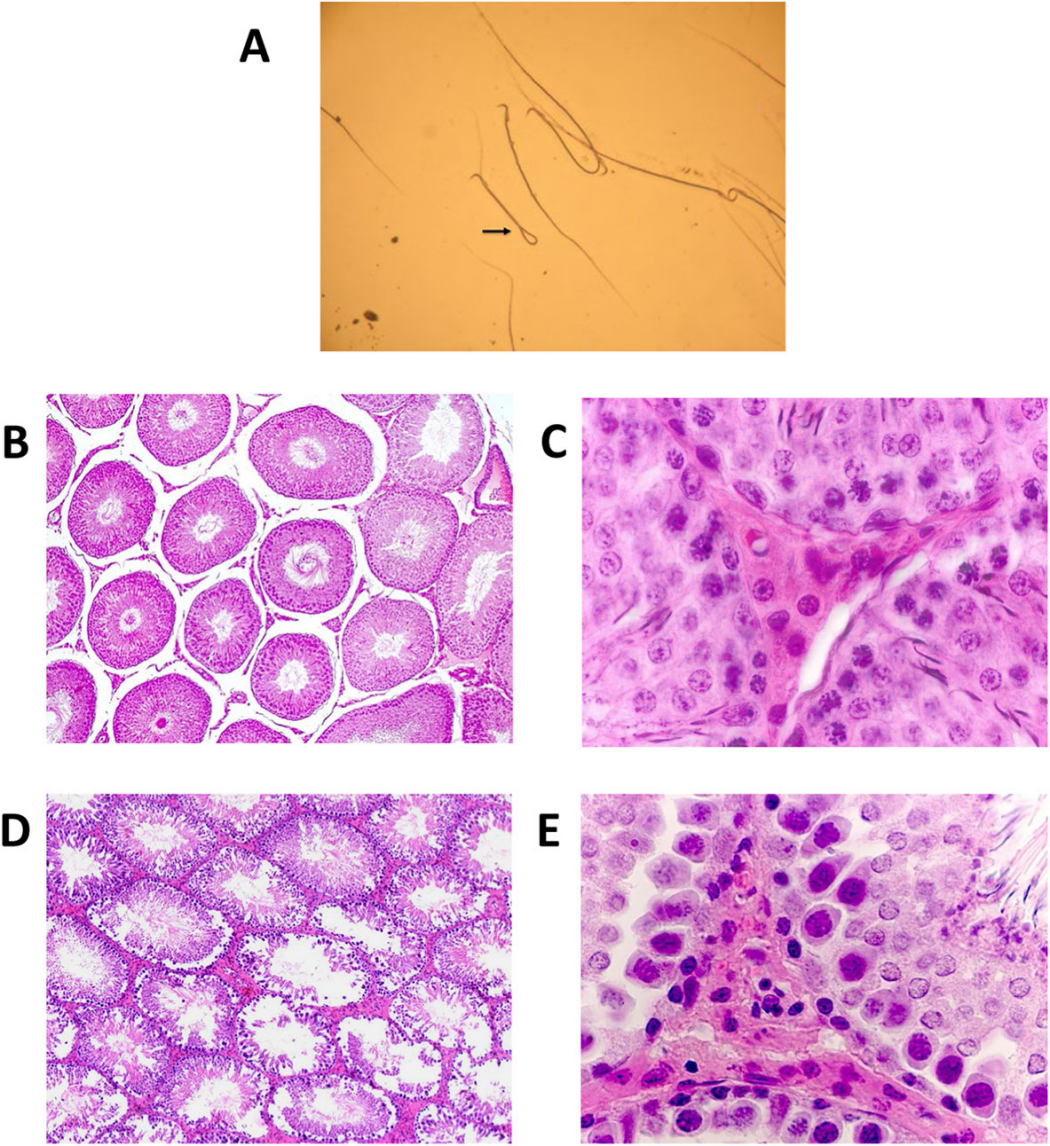
Effects of PADE on sperm formation and testicular structure. **a** A pathological form of spermatozoon in rats receiving PADE 125 mg/kg for 60 days, an arrow indicates a spermatozoon with an attached tail to the head; **b** Testis of control male rat, tubules at different stages of the spermatogenic epithelium cycle; **c** Testicular interstitial tissue of control male rats, large light glandular Leydig cells; **d** Testis of a rat receiving PADE 125 mg/kg for 60 days, tubules at different stages of the spermatogenic epithelium cycle, Disorganization of the germinal epithelium, reduction of tubules with mature sperm; **e** Interstitial tissue of rats receiving 25 mg/kg PADE for 60 days, small pyknotic Leydig cells. Hematoxylin-eosin stain. Magnification B and D × 200 and C and E × 400

Oral treatment of male rats with PADE at a dose of 125 mg/kg caused pronounced effects on male reproductive organs (Table 2). Relative decreases were observed in the total number of spermatozoa produced, but differences were not statistically significant. The decrease in the number of motile spermatozoa in groups receiving low and high doses of PADE and the spermatozoon movement’s duration in the group receiving PADE 125 mg/kg were statistically significant (*p* < 0.05, Table 2). The number of pathological forms of spermatozoa increased 3.4 times in the treated group (*p* < 0.05). The most pathologic types of spermatozoa had the ends of the sperm tail attached to the heads of the spermatozoa (Fig. 1a). Morphologic evaluation of testis from the males treated with PADE 125 mg/kg revealed a statistically significant decrease in the spermatogenesis index.

Figure 1b demonstrates the structure of testicular tubules of control male rats at various spermatogenic epithelium cycle stages. Histological evaluation of testes of treated and control rats revealed that Leydig cells in the testes of control rats are lighter in color with a larger, more rounded or oval in shape nuclei containing clumps of euchromatin and heterochromatin evenly distributed in the nucleus (Fig. 1c). Figure 1d shows the germinal epithelium’s disorganization and reduced tubules with mature sperm in male rats treated with 125 mg/kg of PADE for 60 days. Leydig cells had reduced nucleus diameters and increasing the number of pyknotic nuclei (Fig. 1e). Morphometric analysis of histologic slides determined that PADE treatment had a pronounced dose-dependent effect on Leydig cell nuclei (Table 3).

**Table 3:** Morphometric analysis of Leydig cells

Study Groups^a^	Number of Leydig cell nuclei per section	Median (L 25%; U75%)
Control (H_2_O)	304	24.22 (20.53, 28.33)
PADE, 25 mg/kg	242	21.18 (17.41, 24.27) **
PADE, 125 mg/kg	236	20.22 (17.41; 23.45) **

^a^ Male rats were treated with either H_2_O (control) or PADE (25 or 125 mg/kg) for 60 days before mating; ** *p* < 0.05

### Effects of male treatment with PADE on fetal development

The effect of PADE treatment of male rats on fetal development was examined on day 20 post-fertilization (Table 4). Five to 8 dams were inspected in each group. Repeated treatment of male rats with low and high doses of PADE did not have a significant impact on fetal development. Control and PADE treated groups had approximately the same numbers of the implantations and live fetuses per dam. Fetuses had similar weights.

**Table 4:** Effects of male treatment with PADE on fetal development

Parameters	Study groups^**a**^		
	***Control, H***_***2***_***O***	***PADE, 25 mg/kg***	***PADE, 125 mg/kg***
***Fetal development on day 20 post fertilization (mean*** ***+*** ***SD)***			
No. of dams examined	5	8	8
No. corpus luteum/dam	12.6 + 0.7	11.3 + 0.8	12.3 + 0.6
No. implantations/dam	11.0 + 0.8	9.9 + 0.6	10.5 + 0.6
No. live fetuses/dam	10.2 + 0.7	9.0 + 0.3	9.9 + 0.8
No. resorptions/ dam	0.8 + 0.3	0.9 + 0.5	0.6 + 0.3
Fetal weight, (g)	2.4 + 0.1	2.3 + 0.1	2.3 + 0.1
Pre-implantation death (%)	12.7	12.4	14.6
Post-implantation death (%)	7.3	9.1	5.7

^a^Male rats were treated with either H_2_O (control) or PADE (25 or 125 mg/kg) for 60 days before mating

Interestingly, the treatment of male rats with 125 mg/kg of PADE decreased the number of resorptions per dam and the percent of post-implantation fetal death but increased the percent of pre-implantation death compared to the control group (Table 4). However, the reduction in the number of resorptions and percentages of post-implantation fetal death as well as the increase in pre-implantation death were not statistically significant.

### Effects of male treatment with PADE on offspring development

The effect of PADE on offspring survival and development was examined on day 21 after birth (Table 5). Nine to 12 litters were examined. Matting of intact female rats with males receiving PADE 125 g/kg resulted in increased pups per litter. However, these differences were not statistically significant.

**Table 5:** Effects of male treatment with PADE on offspring development

Parameters	Study groups^**a**^		
	***Control, H***_***2***_***O***	***PADE, 25 mg/kg***	***PADE, 125 mg/kg***
***Offspring survival (mean*** ***+*** ***SD)***			
Number of litters examined	12	9	9
No. offspring/ litter at birth	8.6 + 0.9	9.3 + 0.7	11.3 + 0.6
No. of survived offspring/litter on day 21 after birth, (%)	8.6 + 0.9 (100%)	9.3 ± 0.7 (100%)	11.3 ± 0.6 (100%)
***Offspring behavioral development on day 21 after birth (mean*** ***+*** ***SD)***			
Number of line crossing	45.3 + 1.3	43.8 + 3.3	47.9 + 2.5
Head dipping	12.1 + 0.7	11.8 + 1.3	12.3 + 0,8
Grooming	0.9 + 0.3	0.9 + 0.1	0.8 + 0.2
Defecation	1.4 + 0.3	2.5 + 0.5	2.8 + 0.4
Rearing	0	0.03 + 0.01	0.02 + 0.01

^a^ Male rats were treated with either H_2_O (control) or PADE (25 or 125 mg/kg) for 60 days before mating

**Fig. 2:**
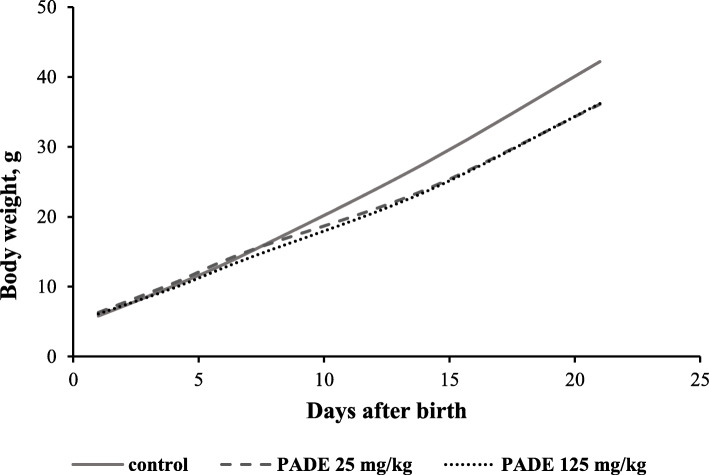
Weight gain by the offspring born from the control male rats and male rats treated with PADE

All newborn rats survived to day 21 after birth. The dynamics of the offspring weight changes in the treatment groups were comparable to the changes in pups’ weights in the control groups (Fig. 2). The control group gained slightly more weight, but differences were not statistically significant.

An open-field test on day 21 after birth evaluated the offspring’s motor-sensory reflexes. All pups from the treated groups demonstrated similar behavioral and locomotive reflexes as their counterparts in the control groups (Table 5). There were no statistically significant differences in the number of lines crossed by pups nor in the grooming and head dipping activities between PADE-treated and control groups. However, there was a minor increase in the rearing and defecation activities in the groups of pups born from PADE-treated fathers. Differences were not statistically significant.

## Discussion

The popularity of medicinal herbs and their accessibility to the general population makes it entirely possible that men and women of childbearing age will use medicinal herbs or botanical drugs. The evaluation of potential drug effects on these individuals’ fertility is an essential part of preclinical studies since this could affect the viability and development of their offspring.

Many medicinal herbs have documented adverse effects on the male reproductive systems of experimental animals. It is known that aqueous or ethanolic extracts from *Aegle marmelos* L. (Indian bael), *Abrus precatorius* L. (rosary pea), *Mimusops elengi* L. (Spanish cherry) exhibit anti-spermatogenic and anti-fertility effects [18–20]. *Curcuma longa*, L., a well-known and widely used herb, demonstrated anti-fertility effects in experimental animals [21]. Treatment of albino rats with aqueous rhizome extract of *Curcuma longa* for 60 consecutive days decreased spermatozoon counts and their motility resulting in reduced fertility [21]. Treatment of Wistar Albino rats with *Carica papaya* L. preparation in doses 100, 250, and 500 mg/kg for 52 weeks induced azoospermia [22]. *Allium sativum* L. extract treatment inhibited the Leydig cell activity [23]. *Enicostemma axillare* (Lam.) Raynal leaf and *Urena lobata* L. (Caesarweed) root extracts inhibited spermatogenesis and steroidogenic activity of Leydig cells [24]. It should be noted that in most cases, these changes were reversible after 50–60 days after treatment withdrawal [24].

The study presented herein study demonstrated that oral, 60-day treatment of male rats with supra-therapeutic doses of the thyrotropic botanical drug, PADE (8 and 40 times exceeding median therapeutic dose recommended for clinical trials), led to dose-depended alterations in male reproductive functions. PADE treatment decreased the number of motile spermatozoa and the time of their movement. PADE treatment decreased the spermatogenesis index and increased the number of pathologic forms of spermatozoa. This study also demonstrated that PADE treatment of male rats for 60 consecutive days reduced Leydig cell nucleus diameters and an increasing number of pyknotic nuclei in these cells. Studies aimed to determine whether PADE effects on the male reproductive system can be reversed in progress.

PADE is a multi-constituent botanical drug. It comprises of catechins, gallic acid, coumaric acid, polysaccharides, phytosterols, and polymeric proanthocyanidins of unknown structure. It has been reported that treatment of mice with gallic acid prevents DNA fragmentation and testicular tissue atrophy induced by cyclophosphamide administration [25]. Green tea catechins have a positive effect on sperm viability, morphology, motility, fertility rate, and gamete quality of experimental animals [26, 27]. The long-term treatment of male rats with β-sitosterol resulted in decreased testicular weight and sperm concentration [28]. A more recent publication investigated the effects of phytosterols on the adrenal and reproductive functions of Japanese male quails [29]. Quail chronically fed with phytosterols significantly reduced testicular weight and testosterone levels. Additionally, it has been reported that phytosterols reduce testosterone levels in male rats [30]. Therefore, it is likely that phytosterols present in PADE are partially responsible for the male reproductive system’s harmful effects. It is also possible that such components of PADE as gallic acid and catechins ameliorate effects of phytosterols on male reproductive system [30].

Our experiments conducted in rabbits demonstrated that PADE had a selective toxic effect on experimental animals’ pituitary glands. This resulted in a decrease in the size of basophilic cells in the adenohypophysis and dose-dependent spermatogenesis inhibition, potentially due to a decreased synthesis and secretion of gonadotropic hormones (unpublished pathohistological data). Based on the data presented above, it can be concluded that it is likely that phytositosterol is responsible for the toxic effects of PADE on the male reproductive system and the observed changes could be in part a result of reduced production of testosterone.

The majority of patients with thyroid dysfunction are female [1, 31]. Our comparative work has investigated PADE effects on the female reproductive system and female PADE treatment on offspring development, and the study determined that PADE does not significantly affect the female reproductive system (manuscript in preparation).

Evaluation of how parents’ treatment with a candidate drug affects fetal and offspring development is also an essential part of preclinical studies. This study has demonstrated that PADE resulted in suppressed spermatogenesis in male rats. However, the amount of remaining healthy sperm was sufficient for fertilization of a similar number of female rats as in the control group. Offspring born from PADE treated males was healthy.

What are the clinical implications of the adverse effects observed in this study? Specifically, should PADE be prescribed to male patients with thyroid dysfunction? The reliability of studies investigating spermatotoxicity in animals was investigated by Elizabeth Rayburn and colleagues [32]. They examined 235 FDA-approved small molecule drugs with spermatotoxic effects, and only 49 had documented negative impact on men’s reproductive health. Notably, 155 medications were determined to be spermatotoxic in animals but had no human data. Authors concluded that current animal studies assessing drug spermatotoxic effects are not particularly good predictors of the drug effect on men’s reproductive health. Currently, we can only speculate whether dose escalation in clinical trials (e.g., median therapeutic dose and dose exceeding therapeutic dose by 5 times) will have similar effect on men’s reproductive system. Potentially, results from this animal study can be used to model the exposure-response relationship and adverse outcomes in humans.

Although many medicinal herbs have transient effects on the male reproductive system, there is a potential risk of impaired spermatogenesis when PADE is used in doses exceeding the recommended therapeutic doses. This study suggests that PADE treatment of reproductive age men should be avoided or recommended with caution. The doses prescribed to these patients should not exceed the therapeutic dose. Treating physicians should consider whether the expected benefits of PADE treatment to the male parent exceed possible risks to male fertility and reproductive health as well as potential effects on fetal and offspring development.

## Conclusions

Oral administration of supratherapeutic PADE to male rats for 60 consecutive days resulted in a decreased number of motile spermatozoa, decreased sperm mobility time, and an increased number of pathological forms of spermatozoa, while did not significantly affect the ability of males to fertilize naïve female rats. PADE administration to male rats did not adversely affect the prenatal development of the offspring born from male rats treated with PADE mated with naïve females. The supratherapeutic treatment of male rats with PADE did not affect offspring survival, weight gain, and motor activity. PADE should be prescribed with caution to men of reproductive age. More studies are needed to determine whether PADE could affect man reproductive functions.

## Data Availability

All data generated or analyzed during the present study are presented in the results section.

## Abbreviations

FDA: Food and Drug Administration
PADE: *Potentilla alba* dry extract
TSH: Thyroid-stimulating hormone
U.S.: United States
VILAR: All-Russian Institute of Medicinal and Aromatic Plants
